# Does plastic waste present an infection hazard? Current evidence, surveillance challenges and research priorities

**DOI:** 10.1186/s42522-026-00237-0

**Published:** 2026-08-13

**Authors:** Patrick Ken Kalonde, Kestern Mkoola, Michael Ormsby, Taonga Mwapasa, Precious Innocent Mastala, Madalitso Mphasa, Katalina Tonthola, Fanuel Meckson Bickton, Peter Nambala, Tony Robertson, Richard S. Quilliam, Kondwani Chidziwisano, Tracy Morse, Christopher M. Jones, Michelle C. Stanton, Peter M. Atkinson, Marc Y.R. Henrion, Nicholas Feasey

**Affiliations:** 1 Malawi-Liverpool-Wellcome Programme, Kamuzu University of Health Sciences, Blantyre, Malawi; 2 Liverpool School of Tropical Medicine, Liverpool, UK; 3 Faculty of Science and Technology, Lancaster University, Lancaster, LA1 4YR UK; 4 School of Biodiversity, One Health and Veterinary Medicine, University of Glasgow, Glasgow, UK; 5 Centre for Water, Sanitation, Health, and Appropriate Technology Development (WASHTED), University of Business and Applied Sciences, Blantyre, Malawi; 6 University of Arizona, Arizona, USA; 7 Department of Rehabilitation Sciences, School of Life Sciences and Allied Health Professions, Kamuzu University of Health Sciences, Blantyre, Malawi; 8 Department of Physiotherapy, School of Therapeutic Sciences, Faculty of Health Sciences, University of the Witwatersrand, Johannesburg, South Africa; 9 School of Health and Wellbeing, University of Glasgow, Glasgow, G12 8TB UK; 10 Biological & Environmental Sciences, Faculty of Natural Sciences, University of Stirling, Stirling, FK9 4LA UK; 11 Department of Civil and Environmental Engineering, University of Strathclyde, Glasgow, UK; 12 The School of Medicine, University of St. Andrews, St. Andrews, UK

**Keywords:** Plastic waste, Antimicrobial resistance, One Health, Environmental surveillance, Remote sensing

## Abstract

Unmanaged plastic waste is widespread in settings with limited waste collection, yet its contribution to infectious disease risk remains poorly understood. In this review, we synthesise current evidence on the human health implications of plastic waste, with a specific focus on environmentally persistent bacteria that are transmitted to humans through the faecal oral route. We examine studies documenting microbial colonisation of plastic surfaces, including pathogenic and antimicrobial-resistant bacteria, and assess how well existing human and environmental surveillance systems capture these risks. We also review recent developments in remote sensing for detecting waste accumulation and evaluate spatial and statistical approaches that could link mapped waste to human exposure or infection data. Drawing on this synthesis, we identify major gaps that limit assessment of plastic-associated disease risk and outline key research priorities: improving methods for mapping waste using remotely sensed imagery, characterising spatial and temporal patterns of waste accumulation, and developing exposure metrics that incorporate environmental transport pathways. This review provides a foundation for advancing One Health research on plastic waste and its potential role in disease transmission.

**Supplementary Information:**

The online version contains supplementary material available at 10.1186/s42522-026-00237-0.

## Background

In many low-income countries, limited capacity for waste collection and safe waste disposal remains a major challenge [1, 2]. This problem is acute in rapidly growing informal or unplanned settlements where communities often fall outside the reach of formal waste management systems, leaving residents with few alternatives but to discard waste into their surrounding environment [3]. As a result, unmanaged waste often accumulates in open spaces, especially along roads, near homes, schools, markets and drainage networks, ultimately forming persistent and unmanaged waste piles [4, 5]. These piles create potentially high-risk zones where human, animal, and environmental health intersect. This issue, thus, represents a One Health challenge, requiring an integrated approach [6, 7]. Yet, despite their scale and persistence, such waste sites remain largely overlooked in urban planning and public health discourse.

**Fig. 1:**
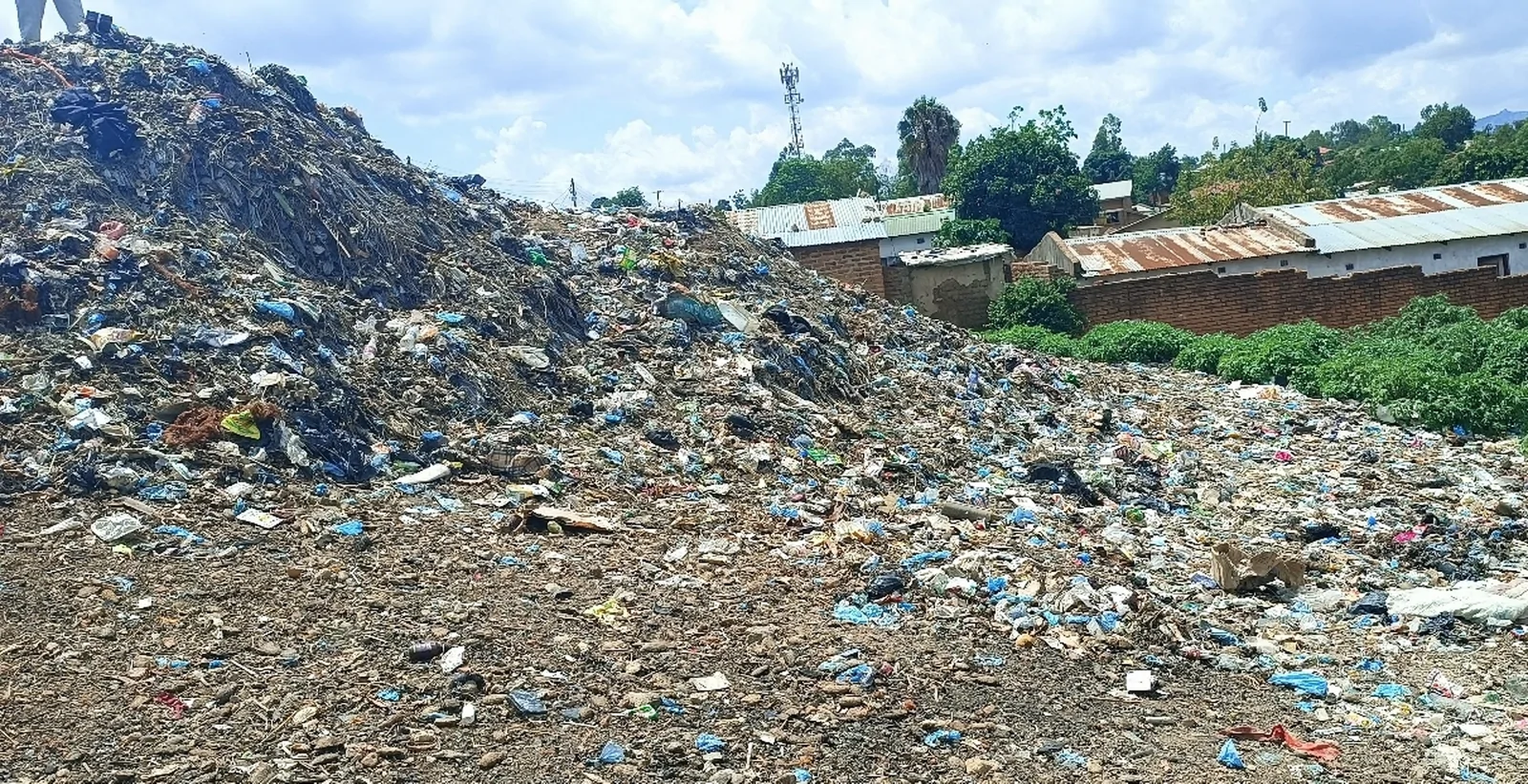
A typical waste-pile in a setting where waste disposal in the environment is practiced. The waste pile includes a mix of waste including plastics (most of them blue plastics which are given free of charge in the country shown, Malawi), food waste, organic waste and numerous other materials. Photograph taken by the lead author

Plastic waste is often the most visible and persistent component of unregulated waste piles (Fig. 1), accounting for approximately 50% of the total surface area in many settings [8]. This dominance reflects a broader global trend: plastic production has surged to an estimate of 459 million tonnes annually (as of 2019), an increase of approximately 2,195% since 1950, when global production was around 20 million tonnes [9]. Much of this plastic comes from single-use packaging, agricultural films (thin plastic sheets used to cover soil or crops to retain moisture and control weeds), food wrappers, personal care containers, and medical disposables, and is typically composed of polymers such as polyethylene (PE), polypropylene (PP), and polyethylene terephthalate (PET).These materials are lightweight, durable, chemically stable, hygienic, waterproof, versatile and relatively inexpensive to produce, qualities that have made plastic an indispensable part of modern life. From households to hospitals, farms to factories, plastics have become deeply embedded in the fabric of everyday living. The problem, however, lies in its afterlife. Plastics are poorly recycled, particularly in low- and middle-income countries (LMICs) where formal recovery systems are limited or absent. In Africa, for example, only about 4% of waste is recovered by informal recyclers [10]. Globally, it is estimated that 79% of plastic waste ends up in the environment after use [11]. Once discarded, plastic undergoes slow degradation through exposure to sunlight and oxygen, breaking down into smaller fragments that persist in soils, water bodies, and even the air, for decades [12, 13]. Its persistence, coupled with its ubiquity, makes plastic waste a growing concern for both environmental and public health.

Although there is growing evidence that plastic waste accumulates in areas with a high burden of infectious disease [14, 15], whether and how this waste contributes to transmission is not well understood, and this review addresses the question by pursuing two connected aims. First, we review the evidence linking plastic waste to human health, considering several proposed pathways, including toxic emissions from open burning, chemical leaching, and the creation of mosquito breeding habitat, before concentrating on bacterial colonisation of plastic surfaces, where evidence that plastic waste can act as a reservoir for pathogenic bacteria is consistently reported. Second, we ask how bacteria-related risks from waste can be identified and tracked, assessing existing human and environmental surveillance methods. We specifically examined a unique role of remote sensing for mapping waste, and spatial statistical methods for linking it to human exposure. The first part of the review therefore sets out why bacterial risk should be prioritised, and the second part builds directly on this by considering how waste mapping and its linkage to human exposure have been approached to date, before setting out a clear research direction for addressing the gaps that remain. Although this review synthesises evidence from a range of settings, several of the observations and examples highlighted are informed by the authors’ experience conducting clinical, environmental, and spatial surveillance research in Malawi.

### Human health risks associated with plastic waste

Plastics are associated with a range of human health risks, but in many low- and middle-income countries the most immediate concerns stem from what happens after plastics are discarded (See Additional file 1: Table S1 for a summary of disposal practices, associated hazards, and possible health outcomes). Open burning remains common where waste services are limited [16], producing $\:{PM}_{2.5}$, dioxins, and other toxic by‑products [17, 18]. These emissions contribute to respiratory inflammation and chronic disease, including asthma, chronic bronchitis and cardiovascular complications [19]. Such exposures typically occur in crowded environments where burning is used to reduce waste volume or supplement household fuel, bringing pollutants into direct contact with the most vulnerable residents.

When plastics accumulate rather than burn, they present a different set of hazards. Additives such as bisphenol A and phthalates [20, 21] leach into surrounding soils and water sources [22], contaminating shallow wells and surface water used for drinking, cooking and washing. This contamination intensifies during the rainy season, when runoff mobilises leachates and transports them into households and shared water points [23, 24]. These chemical risks are well recognised, but they represent only part of the health burden associated with the persistence of discarded plastics in the environments where people live.

Plastic items such as bottles, bags and containers often trap rainwater and create small but persistent aquatic microhabitats capable of supporting *Aedes* and *Anopheles* mosquitoes [25]. Vector‑borne diseases account for a substantial proportion of the global infectious disease burden [26], and mosquitoes require water for larval development at every stage [27]. Evidence from diverse settings links discarded plastics to enhanced mosquito breeding. During the 2000 dengue outbreak in El Salvador, households with water‑holding plastics colonised by mosquito larvae experienced higher dengue incidence [28]. In many other locations, studies have simply documented immature *Aedes* stages developing in rainwater accumulated within discarded plastic containers, including reports from Bangladesh [29], Sri Lanka [30], India [31–33], Tanzania [34, 35], Brazil [36], Canada [37] and the United States [38]. Plastics are one contributor among many artificial containers, and mosquitoes also exploit water storage vessels and other receptacles that may provide more stable breeding conditions [39].

While some plastic‑associated chemicals have also been shown experimentally to affect mosquito development, including shortened emergence times under specific laboratory conditions [40], it is microplastics that have attracted more attention as a potential modifier of mosquito biology. Microplastics (MPs), small plastic fragments now widespread in aquatic environments, are readily ingested by larvae, and laboratory studies have explored their effects on survival [41–43], body weight [41, 43, 44], wing morphology [43, 45], gut microbiome composition [44, 46] and insecticide susceptibility [44]. Most studies report minimal or inconsistent effects on survival and morphology, and it is important to note that many of these effects have been observed at MP concentrations far higher than those measured in typical field settings [45]. MPs can, however, alter larval gut microbiota with plausible consequences for digestion, immunity and pathogen susceptibility [46] and may adsorb insecticidal compounds, reducing their bioavailable concentration [44]. These mechanisms remain under active investigation [47] and, although relevant to the broader ecological context of plastic pollution, they are not considered further in this review.

The most consistent and concerning body of evidence relates to the capacity of discarded plastics to act as microbial habitats [48]. Plastic surfaces readily support multispecies biofilms, often termed the plastisphere, providing stable substrates on which pathogenic bacteria can persist [49]. Pathogens have been identified on plastics collected from peri urban environments [50], estuarine systems [51] and waste piles in informal settlements [52], and some retain virulence after prolonged environmental exposure [53]. Plastics recovered from sewage, soils, beach debris, river systems and dumpsites frequently carry organisms with antimicrobial resistance (AMR) genes [50–52, 54]. Evidence from Ndirande in Blantyre demonstrates that waste pile plastics can harbour antimicrobial resistant bacteria at higher levels during the wet season, when moisture promotes biofilm growth and microbial survival [52]. Horizontal gene transfer within biofilms provides a plausible mechanism for the accumulation of resistance determinants in these communities [55]. These observations indicate that plastics function as persistent environments in which pathogenic and antimicrobial resistant bacteria can persist, interact and amplify, particularly in settings where waste accumulates close to where people live.

Enteric disease remains a leading cause of death worldwide, with a disproportionate impact on children under five [56, 57], and antimicrobial resistance in enteric pathogens is an increasing contributor to this burden, with drug-resistant *Salmonella Typhi* now endemic in several African and South Asian settings [58, 59]. Against this background, the persistence of the plastisphere warrants scrutiny. Current evidence does not suggest that biofilm formation is specific to particular plastic polymers [60–64], and biodegradable plastics also support extensive microbial colonisation [65, 66]. Microbes colonise a wide range of discarded materials, including fabrics and organic waste [52], indicating that material type is not the determining factor. Plastics differ, however, in that they degrade extremely slowly, potentially sustaining microbial communities, including pathogens and AMR genes, for long periods and even as they fragment into microplastics [67]. For this reason, and because microbes show no preference for one substrate over another, large and unmanaged waste piles that contain substantial quantities of plastic should be viewed with particular concern. They create durable and accessible microbial reservoirs that can sustain pathogens, facilitate resistance exchange and support environmental dissemination in settings where waste management is limited. Of the hazards associated with plastic waste, bacterial colonisation therefore presents the clearest and most consistently documented risk to human health: the evidence linking plastics to bacterial persistence and AMR is more extensive than that available for other pathways, and the mortality burden of enteric disease specifically makes this risk pathway a priority for further investigation. The remainder of this review accordingly focuses on bacteria, and enteric bacteria in particular.

### Monitoring and assessing health risk

Plastic waste has been found to harbour enteric and opportunistic bacterial pathogens including *Salmonella* spp., *Klebsiella pneumoniae*, *E. coli*, and potentially pathogenic *Vibrio* spp [50–52, 54, 60, 63, 64, 67–74]. Several isolates recovered from plastic waste exhibited antimicrobial resistance, although not all of members of these genera are pathogenic or AMR associated. Infections caused by these bacteria are responsible for major disease burden in settings with limited sanitation and hygiene, with the majority of cases occurring in South Asia and Africa [75]. Outbreaks linked to multidrug resistant (MDR) bacterial strains have been reported in these regions [76]. Despite growing evidence that plastics harbour pathogenic and antimicrobial‑resistant bacteria, the extent to which waste accumulation influences human exposure remains poorly characterised [77]. Investigation of the microbes associated with waste piles in the environment and exploring their links to humans can help clarify how plastic waste contributes to disease burden and how improvements in environmental conditions might translate into improvements in human health. The following section reviews what is known about the presence of human pathogens in the environment and approaches to human and environmental surveillance (see Additional file 1: Table S1).

#### Surveillance of pathogenic bacterial infections in humans

Surveillance of pathogenic bacteria and associated disease in human populations involves continuous and systematic collection, analysis and interpretation of health-related data [78]. Surveillance can be implemented in healthcare facilities or in the community, and is typically carried out either by Ministry of Health clinical and laboratory staff as part of routine care, or by research institutions running dedicated studies. Long-term, systematic bacteriological surveillance of the kind carried out by research institutions remains uncommon in low-income countries, and where it exists its sustainability and coverage depend on continued research funding rather than routine national systems. Where present, such programmes generate valuable longitudinal data for studying trends in bacterial infections [76]. Surveillance programmes, even in well-resourced healthcare settings typically prioritize monitoring overall prevalence over capturing their geographic distribution at a fine scale. Linking cases to specific places is straightforward only in settings with well-defined postal or addressing systems. Across much of the LMICs, formal address systems remain incomplete; in sub-Saharan Africa specifically, this has been linked to gaps in emergency service delivery and access to basic infrastructure [79]. Although substantial variation is likely to exist between countries and between urban and rural settings within the same country, health facilities often lack standardized conventions regarding the granularity at which patient locations are documented [80, 81]. In the absence of a formal addressing system, an alternative approach involves asking patients to identify their home locations directly on digital maps, an approach implemented by some initiatives [82, 83]. Research programmes can link facility-based records to residential locations or administrative units, enabling mapping of disease patterns across space [84–87]. Such linkages have been used to explore spatial clustering and identify areas of elevated disease burden [88]. However, fine-scale geographical tracking of bacterial infections remains uncommon outside of dedicated research programmes.

A shortcoming of passive surveillance systems is their dependence on healthcare facility records that only capture those who seek care, by definition, they overlook asymptomatic carriers or paucisymptomatic individuals who do not seek health care [89–92]. This limitation is compounded by low rates of care-seeking itself. Across low- and middle-income countries, only around a third of diarrhoeal cases result in a visit to a hospital or clinic [93]. Barriers including distance to facilities, transport costs, and limited trust in or availability of care have been identified consistently across settings [94]. Passive surveillance therefore substantially undercounts disease burden, particularly among populations facing the greatest access barriers, such as those in peri-urban informal settlements. Such individuals may nonetheless act as reservoirs and contribute to ongoing transmission and, therefore, represent important targets for public health intervention, particularly during outbreaks. Surveillance can also be extended to apparently healthy individuals through community-based studies, however these “active” surveillance systems are more expensive [90]. These studies collect biological samples such as blood, stool, or urine [95–97], and in some cases, record point-level information such as GPS coordinates of the participants’ households [96]. These samples may be analysed using the same tests as those collected in healthcare facilities. Alternatively, serological assays can be employed to detect pathogen-specific antibodies, potentially (subject to the test parameters) capturing clinical, subclinical, and past infections. While the use of serology to quantify exposure in asymptomatic individuals was common during the COVID-19 pandemic, a single blood sample can be used to test for antibodies against multiple pathogens [98–100]. When linked with participant locations, serological data offer an opportunity to map geographical patterns of human exposure to pathogenic bacteria and to explore associations with waste, thereby strengthening surveillance.

#### Environmental surveillance of pathogenic bacteria

Environmental surveillance of pathogenic bacteria aims to determine where the bacteria occur in the environment, and how it persists under different conditions. It focuses on detecting and quantifying pathogens in environmental sources such as animals [101, 102], waste piles [52], soil [103, 104], surface water [105], wastewater [106] and river water [106–108]. Environmental samples are usually collected and transported to laboratories to isolate target bacteria using culture-based methods [71, 73, 109]. Isolates are then confirmed and characterised using antibacterial susceptibility testing or molecular techniques such as quantitative PCR [71]. Environmental surveillance is a relatively low-cost approach for identifying circulating pathogens and strains [110], particularly in settings lacking systematic clinical surveillance [111, 112], and it poses fewer ethical and logistical challenges because individual-level consent is generally not required [113]. In contexts marked by inequality, it can capture information from areas poorly served by formal health systems or research teams [114] and has been recommended for integration into broader AMR monitoring programmes [115].

This general approach to environmental surveillance is not new, and has been applied to a wide range of enteric pathogens for decades, including *Vibrio cholerae*, rotavirus, hepatitis A virus, and non-polio enteroviruses, well before it became associated with the COVID-19 pandemic [116]. A systematic review of 100 pre-pandemic studies across 38 countries identified 25 distinct pathogen families under environmental surveillance [116]. Much of the field’s current methodology, however, was shaped specifically by global poliovirus eradication efforts [117–119], where environmental data guided vaccine strategies and assessed intervention impact [116]. More recently, wastewater surveillance gained prominence during the COVID-19 pandemic as a cost-effective method for tracking community infection trends [120–122], and has since expanded further to a wider array of viral, bacterial, and parasitic targets [123]. However, these approaches have largely relied on centralized wastewater infrastructure, which is limited or absent in many low- and middle-income countries, including much of sub-Saharan Africa [124]. In these settings, pit latrines are commonly used instead of sewer systems, and sometimes latrine contents may enter open drains and rivers through direct disposal, flooding, or other events that mobilise waste from containment systems [125, 126].

In settings without formal waste collection, domestic waste is discarded into the surrounding environment [4, 127, 128], creating alternative pathways for pathogen dissemination. Plastics compound this challenge as they are easily transported by wind or rainwater [129], can carry viable bacterial pathogens [68] for extended periods [53, 130], and are increasingly recognized as environmental vectors in their own right [131], yet their dispersal patterns remain poorly quantified. This mobility has significant implications for surveillance. A piece of plastic may carry its associated pathogens well beyond the point at which they were originally acquired, such that surveillance directed at a fixed point of origin is likely to underestimate the associated risk substantially. What is of greater relevance, therefore, is the location at which the plastic, and the pathogens it carries, ultimately accumulate Spatial epidemiology follows the same principle at the population level. Rather than attempting to reconstruct the transmission history of individual cases, it seeks to determine whether disease occurrence or pathogen exposure exhibits spatial clustering and whether these patterns are associated with environmental hazards, reservoirs, or pathways of dispersal across the landscape [132]. Establishing where waste accumulates, and how this relates to patterns of human exposure, therefore constitutes a research priority. It is in this respect that the spatial sciences, encompassing both remote sensing and spatial statistics, assume particular importance. Remote sensing provides a means of mapping waste accumulation at scale. Fine-resolution aerial imagery, including that acquired by drones, may be used to train pattern-recognition models for the detection of waste [133–135], thereby supporting repeated mapping of waste accumulation and dispersal over time. Spatial statistical methods can subsequently be applied to test the association between mapped waste and human exposure, both of which are addressed in the sections that follow.

### Remote sensing for monitoring of waste

**Fig. 2:**
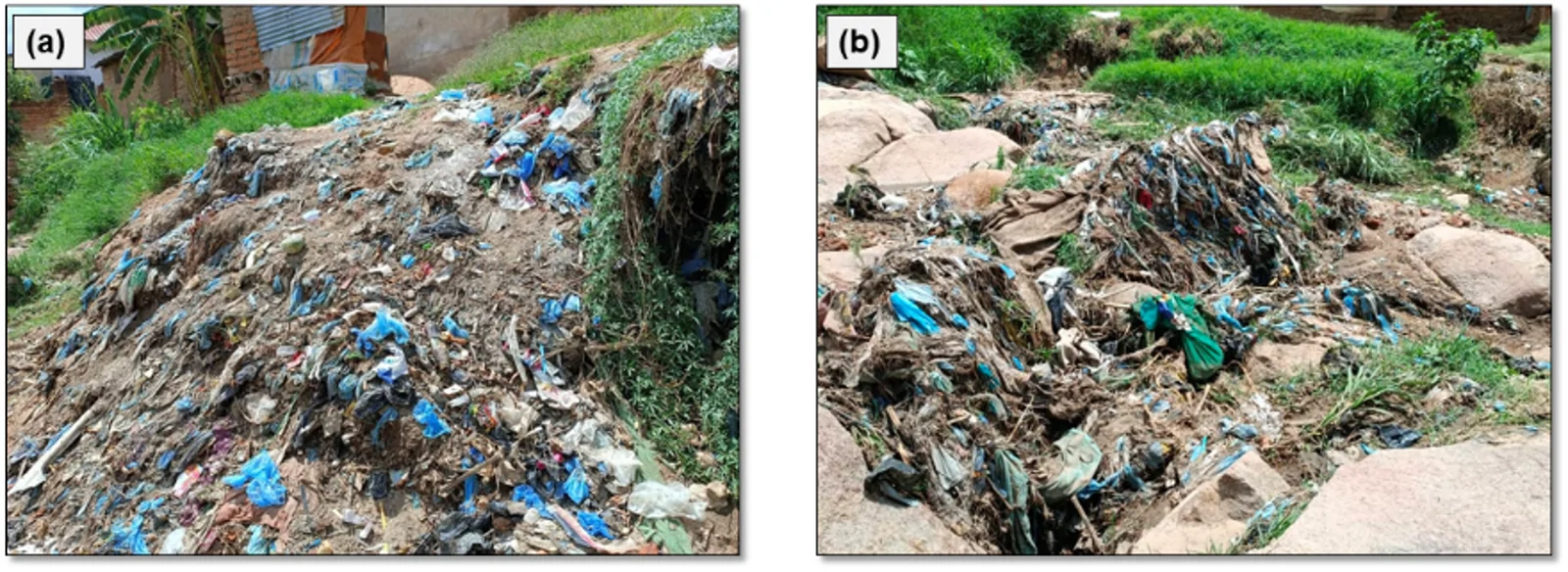
Waste pile dispersal: (**a**) waste accumulating into a pile before rainfall; (**b**) waste pile being dispersed by storm water

Thus far, we have considered waste at the microscopic scale, focusing on pathogens that may pose risks to human health. Waste piles can act as reservoirs of pathogenic microbes, and it is now necessary to “zoom out” and consider the urban and peri‑urban environments in which these piles accumulate and move. Identifying where waste builds up, where it is dispersed by storm water, and where it subsequently re‑accumulates (Fig. 2) is essential for understanding potential exposure pathways. Remote sensing provides a means to capture these spatial patterns by using sensors mounted on satellites, aircraft, or drones [136] to collect spatially continuous observations of reflected light, enabling the mapping of waste across large areas and offering information that would otherwise be unavailable. This capability is particularly valuable in settings with limited formal environmental monitoring systems, where ground‑based observations are sparse or inconsistent.

#### Trends in environmental remote sensing

Remote sensing has evolved from black-and-white aerial photography to full-colour digital imagery captured from controlled angles by drones [134, 137, 138], aircraft [139–141], and satellites [142]. Early aerial photograph images required manual interpretation, but digital acquisition and advances in digital image processing and artificial intelligence now enable automated extraction of key information. A substantial body of research has demonstrated the utility of automated approaches for environmental mapping, including applications such as forest loss mapping [143] and tracking of urban expansion [144]. In contrast, environmental waste assessment has traditionally relied on field surveys centred on manual counting and weighing of waste materials [145], and in some cases, on characterizing the microbial composition of waste [52]. More recently, there has been growing interest in using aerial imagery to locate and quantify waste accumulating in the environment [133, 142, 146]. However, to advance environmental surveillance of waste, including plastic waste, the central question is: how can aerial imagery captured through remote sensing best support the mapping of waste, and potentially improve environmental and/or human disease surveillance? The following sections describe the characteristics of remote sensing data previously used for waste mapping, and with a specific focus on spatial and spectral characteristics.

#### Aerial data: spectral characteristics

Plastic waste can be identified in aerial imagery through its unique spectral properties. While the human eye perceives only visible light (blue: 450–485 nm; green: 500–565 nm; red: 625–750 nm), aerial sensors capture a broader spectral range, including the near- and shortwave-infrared regions, which can reveal materials that are visually indistinct. Laboratory studies show that plastics exhibit distinct reflectance values in the shortwave infrared (SWIR) region [139], and in some cases, the spectra can even differentiate polymer types [147]. Although capturing the unique SWIR region is not yet part of standard aerial imagery, these observations have the potential to guide the design of pragmatic sensors for mapping plastics accumulating in the environment. The thermal properties of plastics have been explored, but their utility remains limited because many surface materials share similar thermal spectra [148]. In contrast, multispectral satellites such as Sentinel-2 have demonstrated the ability to detect plastic waste in coastal environments, for example, in Scotland [149] and Greece [150]. Furthermore, benchmark datasets such as MARIDA have accelerated the development of models for mapping marine litter [151]. Comparable datasets for mapping waste in terrestrial and urban settings are lacking and need to be developed.

##### Aerial data: spatial resolution

Identification of plastic waste from aerial images depends on the spatial resolution of the aerial data [8, 152]. Spatial resolution refers to the smallest discernible unit in an image. Satellite and aerial platforms collect imagery at a wide range of spatial resolutions. Landsat provides imagery at approximately 30 m resolution [153], Sentinel-2 offers a 10 m resolution [154], and Planet Labs’ medium-resolution constellations acquire imagery at about 3 m resolution [155]. Commercial systems such as Pleiades [156] and Vantor (formerly Maxar) [157] provide sub-metre detail. Revisit times range from daily for systems such as Planet Labs [155] to every 16 days for Landsat [153]. Access to the imagery also differs. Sentinel-2 and Landsat data are freely available. Planet Labs provides some imagery for free to educational and research programmes, while fine-resolution products from Planet, Pleiades, and Vantor are commercially licensed. Some satellites can also be tasked to acquire imagery on specific dates. Most optical satellite imagery is affected by cloud cover, whereas radar systems such as Sentinel-1 SAR can penetrate clouds. This limitation has encouraged the use of optical sensing via drones, which enable flexible, cloud-free observations.

Drones can capture imagery with sub‑centimetre detail and can do so flexibly and repeatedly, with the spatial resolution determined by the sensor and flight altitude [8, 152]. Low‑altitude flights at about 10 to 20 m have been used to produce imagery in which individual plastic items were identifiable [158–165]. Although such high‑resolution imagery can be visually interpreted, manual interpretation is labour‑intensive and difficult to scale [166]. As a result, pattern‑recognition methods are increasingly used to automate mapping, with studies showing success in detecting waste along beaches [134, 158, 159, 165, 167, 168] and in open water [138, 140, 146]. These settings have relatively uniform backgrounds, but in urban and peri‑urban areas in low‑ and middle‑income countries where waste disposal is common, very low flight altitudes are impractical because of presence of large trees, buildings, power lines and variable terrain. In these areas, waste accumulates in piles, and mapping such piles is important despite limited studies demonstrating how to map them effectively [133].

Since operating drones at very low altitudes might be unsafe and impractical in urban and peri‑urban areas, it is important to investigate whether imagery collected at higher altitudes, with correspondingly lower spatial resolution, can still preserve the spatial texture required to reliably distinguish waste piles. Waste piles in informal settlements typically consist of heterogeneous mixtures of materials such as soil, organic matter, and plastics rather than isolated items [169], may exhibit spatial patterns that remain detectable even when individual objects cannot be resolved. Human interpreters may recognise these textural cues intuitively; assessing how consistently they do so is a necessary first step. Cohen’s κ is used for agreement between two raters, whereas Krippendorff’s $\:\alpha\:$ extends to more than two, and better accommodates varying resolutions and unclassified patches [170]. Reliable manual interpretation also provides a reference against which automated methods can be evaluated. Automating waste detection is likely to require models specifically trained to capture these textural patterns. Our own work using machine learning to identify waste based on groups of similar pixels suggests that this approach is promising [133], although further refinement is needed, including exploring deep learning methods for waste mapping. Detecting a waste pile and delineating its boundary are distinct aspects of performance. Precision and recall capture false positive and false negative rates, F1 balances the two, and Intersection over Union measures how closely predicted boundaries match reference ones. Reporting these across resolutions and sensors reveals the scales at which each deteriorates, which need not coincide.

#### Techniques for automatic mapping of plastics from aerial imagery

Automatic mapping of plastic waste from remotely sensed imagery has been performed using various automated approaches, which can be grouped broadly into three categories: index-based, machine learning, and deep learning techniques. Index-based methods combine spectral bands using mathematical formulae to highlight and identify plastic waste, and these indices have been developed for uniform environments such as ocean surfaces [142, 171, 172]. Machine learning and deep learning are approaches that use identified examples of waste, allowing the algorithms to learn patterns associated with these examples and optimize the model to maximize the chances of correctly identifying waste. These approaches rely on training data, which are usually generated by manually digitizing waste to indicate its presence or outline [133, 137, 140, 159, 161, 165]. Such trained models reduce reliance on labour-intensive manual mapping [133, 141]. While traditional machine learning methods (such as random forest) require the pre-selection of spectral and textural features [173], deep learning architectures utilize multi-layered neural networks to automatically extract hierarchical patterns directly from the data [174], which might be important for mapping waste. Although machine‑learning and deep‑learning methods show promise for automating waste‑pile mapping, their reliability is limited by the highly variable material composition of waste [133], so future research must develop more robust classifiers that can handle this heterogeneity and generalize effectively across diverse environmental contexts.

### Linking environmental waste to human exposure to bacteria

Given the consistent evidence that bacteria readily colonise plastic surfaces, form persistent plastisphere biofilms and carry AMR genes, and in view of the substantial clinical burden posed by drug resistant bacterial infections [175, 176], the bacteria detected on plastics warrant close attention. The organisms repeatedly isolated from discarded plastics include *Vibrio cholerae*, *Salmonella enterica*, *Escherichia coli* and *Klebsiella* species. These pathogens are carried in the gastrointestinal tract and share a faecal-oral transmission pathway. At present, the strongest biological rationale for a link between environmental plastics and human health lies in the faecal-oral transmission of these enteric bacteria that colonise plastic surfaces, with the oral component reflecting human ingestion of organisms mobilised from contaminated waste. Although rodents and other pests on these waste piles can carry these pathogens to humans [177–179], the more likely and direct routes of exposure in informal urban settings involve contact with contaminated surfaces or the ingestion of water that mobilises bacteria from waste piles. At a basic level, therefore, it is important to determine whether waste sites or waste piles are associated with exposure to these pathogens in a way that allows assessment of whether the burden of enteric bacterial disease is linked to environmental plastics. This remains a notable gap. Establishing such a relationship would clarify whether reducing waste accumulation could contribute to measurable reductions in infection burden. The following section outlines how such linkages can be examined and how spatial hotspots associated with waste can be identified, analysed and interpreted.

#### Conceptual need for linking environmental sources and human exposure

Historically, bacteria have been identified at species level, which limits the information available for understanding transmission dynamics. The advent of whole genome sequencing offers vastly increased resolution to define whether two bacterial isolates are genetically distinct or nearly identical. Ideally, the link between environmental waste and human infections should be established by comparing the genome sequence of pathogens detected in environmental samples with those found in clinical isolates or among asymptomatic individuals [180]. When human strains closely match environmental strains, it provides strong evidence of a flux between human and environmental compartments, although inferring the direction of transmission is yet more complicated [180–183]. Genomic analyses have been used to infer transmission networks by identifying highly similar *E. coli* strains and antimicrobial resistance determinants shared across humans, animals, and household environments, revealing hundreds of cross-compartment transmission events [184]. Apart from genomic similarity, these strains can also be examined in relation to geography; previous research in Blantyre showing that typhoid cases occurring close to one another and within the same river catchment often shared highly similar strains [88], suggesting that river systems may act as conduits for transmission. Nonetheless, mapping exposure populations alongside environmental features such as waste piles or river networks can reveal exposure hotspots and generate hypotheses about transmission routes, enabling targeted interventions to reduce risk. The relationships underlying such mapping are unlikely to hold uniformly across settings. The presence of waste piles, bacterial colonisation and its pathogenicity, the potential for water contamination, and the potential for human contact each depend on local geography, stormwater management, seasonality, and behaviour, and need not generalise beyond the setting in which they were derived. Transferability to other contexts should not be assumed, unless the model has been specifically developed to be sensitive to such geographic variation.

#### Methods for exploring associations across space

Matching strains in environmental and human samples indicates connectivity but not direction; contamination can flow from people to environment (shedding), from environment to people (exposure), or both. For exposed populations, it is important to recognize that not all exposures lead to illness [185], meaning many exposed individuals are never captured in clinical case data. Geotagged disease cases can be aggregated at area level and aerial level models have been used previously to assess whether a high case-load relates to a high density of waste [86]. Whether exposure progresses to disease depends on factors such as pathogen dose and host immunity [186, 187]. Geotagged serological datasets may help bridge this gap by detecting markers of recent (IgM) or past (IgG) exposure, and they hold the potential to provide a more complete picture of where exposure occurs. It should be remembered that bacterial serological markers are intrinsically complex and this science continues to evolve.

On the environmental side, sources of pathogens may include waste piles [52], and their locations can be mapped [133]. When these datasets are combined, several point-level approaches can be used to test whether patterns of human exposure relate to mapped waste accumulation. These include statistical measures of association, where distance to waste is treated as a covariate, spatial clustering techniques, point-process models and geostatistical approaches.

#### Distance-based statistical associations

Statistical measures of association use distance to waste as a way to approximate the likelihood of exposure by assuming that interaction with waste is linked, in some way, to how close an individual is to a waste pile; for example, individuals who live nearer to waste piles are more likely to interact with them and are therefore more likely to be exposed. Because direct observational data on human-waste interactions are rarely available at scale, proximity might serve as a pragmatic proxy for potential exposure. In practice, this involves calculating the straight‑line distance between each individual and the nearest waste pile [86], and incorporating this value into statistical models to examine whether the probability or intensity of exposure‑related outcomes, such as geotagged serological markers of bacterial exposure, varies with distance. Continuous outcomes, including antibody concentrations, can be analysed using linear regression, generalized linear models [188], or random‑effects models for repeated measurements [189], while binary outcomes such as seropositivity can be evaluated using logistic regression or mixed‑effects logistic models. This distance‑based approach provides a simple initial approach for testing whether closer proximity, and therefore greater opportunity for interaction with waste piles, is associated with bacterial exposure, and it can help generate picture to inform targeted interventions. This approach has several limitations, however. Straight-line distance assumes exposure risk declines uniformly in all directions from a waste pile, but terrain, drainage, and the routes people actually walk to reach water, markets, or schools are not accounted for. Waste piles are also treated as static points, when in reality they grow, shrink, and disperse with rainfall and human activity, so a distance measured at one time point may not reflect exposure accumulated over the study period. Results are also sensitive to how distance is modelled and which covariates are included. Most importantly, association is not causation, and a significant distance effect does not establish that waste itself is the source of exposure, given the multiple, overlapping sources of contamination discussed earlier in this review.

#### Spatial clustering approaches

While a variety of spatial clustering methods exist, only a few are suitable for exploring the relationship between population exposure to pathogenic bacteria and environmental waste. Most clustering approaches, such as density-based clustering [190] and Kernel Density Estimation (KDE) [191, 192], are useful primarily for detecting spatial clusters but do not allow formal testing of associations with environmental drivers. In contrast, Kulldorff’s Spatial Scan statistic searches for clusters by sliding circles of different sizes across the study area and evaluating each of these for unusually high case concentration [193]. This approach allows formal hypothesis testing by including covariates such as distance to waste in the model. Local Moran’s I [194], and Getis-Ord Gi* [195, 196] detect statistically significant spatial hotspots, providing confidence that observed clusters are unlikely to have arisen by chance. Although they do not provide a formal link with waste, a corresponding hotspot map of waste points can be generated, and the relationship between pathogen exposure and waste can then be assessed by examining spatial correlation or the degree of hotspot overlap. This overlap does not prove that waste is the direct cause of exposure. Waste and disease may both be linked to the same underlying factors, such as poverty, population density, or poor access to sanitation and water, and these shared factors alone can produce a spatial correlation between waste and disease even without waste itself being the source of exposure. Such analyses can therefore only suggest possible exposure routes, not confirm them; doing so would require accounting for these other factors or gathering exposure data directly from individuals.

#### Point-process models

Spatial point process models provide a robust framework for testing whether human exposure to pathogens is associated with waste locations. In this context, individuals with serological markers of recent or past exposure (e.g., IgM or IgG) or binary infection status are treated as marks on spatial points (i.e., individuals), while waste piles are represented as fixed environmental features. The intensity of exposed individuals can be modelled as a function of proximity to waste, with distance-to-waste included as a covariate in inhomogeneous Poisson [197] or Cox process models [198]. This allows for testing whether exposure is more likely near waste sites than would be expected by chance, while accounting for the underlying spatial heterogeneity. When individual-level outcomes are continuous (antibody concentrations), marked point process models can incorporate these “marks” to assess how exposure levels vary with distance to waste. Additionally, second-order summary statistics, such as Cross-*K* functions or pair correlation functions, can detect clustering of exposed individuals around waste points beyond random expectation.

#### Geostatistical approaches

Exploration of associations using Geostatistics is similar to the statistical approaches mentioned previously, where exposure risk can be modelled as a function of proximity to waste included as a covariate. The key difference is that geostatistical models explicitly account for spatial dependence by incorporating a spatial covariance structure and spatially correlated random effects [199]. This allows adjustment for autocorrelation in the residuals among nearby individuals and enables predictions at unsampled locations through techniques such as Kriging [200]. These models can integrate continuous environmental surfaces and provide uncertainty estimates for predicted risk, offering a more nuanced understanding of the relationship between exposure and environmental hazards.

#### Limitations of proximity and the need for improved exposure metrics

Understanding human exposure to bacteria associated with waste is complex, and simple proximity measures do not fully capture how exposure occurs in real environments. While Euclidean distance offers a convenient way to represent how close individuals are to waste [86], it assumes that those who are nearer are proportionally more likely to be exposed. However, emerging evidence shows that pathogenic bacteria on waste materials can be transported by rainwater and surface runoff, with clustering of infections documented in downstream areas and, in some cases, directly linked to hydrological dispersal of contaminated waste [201]. These observations suggest that proximity alone may inadequately describe exposure risk.

Building on this, it is increasingly evident that exposure is shaped not only by how close people live to waste but also by the environmental pathways that move bacteria through the landscape. Direct interactions with waste piles certainly occur in many settings [202], yet hydrological processes can create additional exposure routes that operate independently of these behaviours. Downstream households, in particular, may be exposed through storm‑water driven transport of waste or through contamination of river water with bacteria shed from plastics [203]. In areas where piped water supplies are unreliable, river water is often used for domestic purposes [204], and such use has been linked with bacterial infection risk [205]. These hydrological pathways can amplify or redistribute exposure in ways that simple Euclidean cannot represent. For this reason, exposure metrics need to account not only for proximity but also for environmental transport processes such as terrain and drainage patterns, which influence where contaminated runoff is likely to accumulate. Incorporating these features provides a more realistic representation of potential exposure pathways and strengthens the basis for subsequent spatial analyses.

Both distance-based and hydrologically-informed exposure metrics remain silent on how people actually interact with waste. In many communities, residents actively sort through waste piles to recover materials for construction or resale; children are commonly observed playing in and around waste piles; and waste piles are frequently crossed as part of routine daily movement, for example to reach water points or markets. None of these behaviours would be captured by distance to the nearest waste pile, nor by models of hydrological transport, both of which treat exposure as a function of location rather than of what people actually do at that location. Capturing this would require direct behavioural or social science data, for example through structured observation or time-activity studies. Such methods carry their own limitations, including observer effects and the difficulty of capturing infrequent but high-risk interactions, but they offer a route to distinguishing active contact with waste from passive proximity to it, and represent an important complement to the spatial approaches discussed above.

### Future research

The scientific community has established that discarded plastics readily develop a “plastisphere” [49], a biofilm capable of harbouring pathogenic and antimicrobial‑resistant bacteria [60]. Earlier discussions of waste‑related health risks also considered the possibility that plastics might influence disease transmission through vector‑borne pathways. However, as summarised in this review, the evidence for mosquito‑related mechanisms is inconsistent, highly context dependent, and often derived from controlled experimental studies whose real‑world relevance is uncertain. In contrast, evidence that plastics act as stable substrates for pathogenic bacteria [52, 53, 74, 130] and some with antimicrobial‑resistant genes [50–52, 54]. This evidence is stronger and more consistent. Much as we know that waste function as an environmental reservoir for harmful microbes, we still lack a clear understanding of how it accumulates in the environment, how it persists and moves across space and time, and how these patterns relate to human exposure. Given the widespread presence of unmanaged waste, particularly in low‑ and middle‑income countries [206, 207], and discovery of drug resistance pathogenic bacteria within waste piles accumulating in these settings [52], an important question arises: should waste accumulation be considered not only an environmental management issue but also a public health concern? Addressing this question requires first understanding how waste in the environment contributes to human exposure to pathogenic bacteria. Progress in this area will depend on detailed examination of where waste builds up, how it changes over time, and how these spatial and temporal patterns intersect with human activity. A clearer understanding of these dynamics will create opportunities to investigate exposure pathways and support the development of interventions that interrupt transmission. To advance this work, research should focus on three priorities:

## Develop robust methods for mapping waste using remotely sensed imagery

Existing pattern-recognition methods have been validated on beaches and open water, where backgrounds are relatively uniform; no validated method exists for the heterogeneous, mixed-material waste piles typical of informal settlements. Advances in remote sensing have made it possible to acquire fine‑resolution aerial imagery, and parallel developments in pattern recognition offer algorithms capable of detecting visual patterns in complex environments. However, waste piles often contain a mixture of plastics, organic matter, and other debris, and we still do not know have pattern recognition models for mapping these waste piles from remote sensing imagery. Research is needed to adapt existing pattern recognition algorithms and train and evaluate models for mapping waste. This work will provide foundational tools for systematically mapping waste from remotely sensed images and enabling establishment of spatial distribution of waste accumulation.

## Observe and explain spatial and temporal patterns of waste accumulation

No study has tracked how waste piles form, persist or disperse over time in these settings, in contrast to the established literature on beach and marine litter dynamics. Although many studies describe the presence of unmanaged waste in developing‑country settings, little is known about how waste accumulates, persists, and disperses across space and time. Waste may be redistributed by human activity, rainfall, flooding, blocked drainage, or informal burning, yet the mechanisms shaping these patterns remain poorly understood. Research should characterise patterns of waste accumulation, and determine which areas are most likely to develop persistent accumulations that may serve as reservoirs for microbial communities. Understanding these patterns is essential for identifying where risks may be concentrated and how patterns of waste accumulation may influence potential exposure.

## Develop novel methods for assessing human exposure to bacteria associated with waste

Distance measures capture neither hydrological transport nor active human contact with waste, as set out above; this priority addresses both. Existing spatial methods for analysing exposure are well established, but they were not designed to capture the epidemiological pathways that are most relevant for waste‑associated bacteria. Simple proximity measures, such as straight‑line distance, overlook the fact that waste and its associated pathogens can move through the environment. Hydrological processes, including storm water runoff, can transport waste materials and pathogenic bacteria downstream and far from their original location [208], creating exposure pathways that do not follow Euclidean distance. Evidence of infection clustering near rivers [88] and in downstream communities [201] further demonstrates the importance of these processes. Exposure is also shaped by how people actively engage with these water sources. Activities such as bathing, washing clothes, and collecting water for household use bring people into direct contact with rivers, drains, and other waste-affected water bodies, and the frequency of such contact varies with age and household role, with young children and women, who more often carry out water collection and washing tasks, potentially facing more frequent and direct exposure. There is therefore a need to evaluate how well current spatial approaches represent real environmental transport mechanisms and to develop new metrics that incorporate hydrological connectivity, and behavioural data on human-water and human-waste interaction, stratified by age and activity, and associated dispersal of pathogens. These approaches will help improve our understanding of spatio-temporal patterns of human exposure to pathogenic bacteria, and guide the design of more targeted One Health interventions.

### Concluding remarks

Unmanaged waste, particularly plastics, is an emerging public health concern that has received limited attention in terms of the human infection risk they present. Plastics provide surfaces for microbial growth and may support the persistence and spread of antimicrobial resistance, yet their role in human infection is not well understood. Although pathogenic bacteria on waste are increasingly considered in environmental surveillance, there are no established methods to assess the health risks they pose. Remote sensing offers a promising way to map waste accumulation, but most applications have focused on beaches and marine environments rather than settings with inadequate waste management such as informal settlements. This review makes three contributions in response: it brings evidence on plastic-associated bacterial risk, the state of surveillance practice, and advances in remote sensing together for the first time within a single One Health framework; in doing so, it identifies a specific gap between the ability to map waste and the ability to link it to human exposure; and it sets out research priorities intended to apply beyond the specific settings from which much of the evidence is drawn. The conditions we describe, constrained waste collection, unregulated latrine emptying, and the resulting mixing of pathogens from multiple environmental sources, are documented broadly across informal settlements in the LMICs, and the priorities identified are intended to generalise accordingly. Developing automated detection tools, studying patterns of waste dispersal, and identifying factors that influence these changes are essential to mapping and characterizing the patterns of waste accumulation and dispersal. Once this knowledge is established, new approaches to link human exposure to pathogens on waste through long-cycle transmission pathways should be developed to strengthen environmental surveillance and guide targeted interventions in resource-limited contexts.

## Supplementary Information

Below is the link to the electronic supplementary material.

Supplementary Material 1

## Data Availability

No datasets were generated or analysed during the current study.
