# Supplementary material for: Does plastic waste present an infection hazard? Current evidence, surveillance challenges and research priorities
Source: One Health Outlook. 2026 Aug 13;8:41. doi: 10.1186/s42522-026-00237-0 (PMC13491761; doi:10.1186/s42522-026-00237-0)
Supplement: Supplementary file 1 [file 42522_2026_237_MOESM1_ESM.docx]

**Appendix 1:** Human health risks associated with different plastic waste disposal practices. The table summarizes the principal hazards, exposure or transmission mechanisms, and the possible human health outcomes.

| **Plastic disposal practice** | **Main hazard** | **Exposure or transmission mechanism** | **Health outcome** |
| --- | --- | --- | --- |
| Open burning of plastic waste | Air pollutants (PM_2.5_, dioxins, furans, volatile organic compounds) | Inhalation of smoke and toxic combustion products | Respiratory inflammation, asthma, chronic bronchitis, cardiovascular disease |
| Open dumping and unmanaged waste accumulation | Leaching of plastic additives (e.g. bisphenol A, phthalates) | Contamination of soil, groundwater and surface water used for drinking, cooking, and washing | Potential endocrine disruption and other chronic toxic effects |
| Open dumping of containers that retain rainwater | Mosquito breeding habitats | Water accumulation in discarded bottles, bags, tyres and containers supports *Aedes* and *Anopheles* breeding | Increased risk of dengue, malaria and other mosquito borne diseases |
| Microplastic contamination of aquatic habitats | Microplastic ingestion by mosquito larvae | Alteration of larval gut microbiome, insecticide susceptibility and development | Possible indirect effects on vector competence; ecological significance remains uncertain |
| Unmanaged plastic waste piles | Plastisphere formation (microbial biofilms) | Colonisation of plastic surfaces by pathogenic bacteria | Increased environmental persistence of enteric and opportunistic pathogens |
| Plastic waste contaminated with faecal material | Antimicrobial resistant bacteria and resistance genes | Biofilm formation promotes persistence and horizontal gene transfer; subsequent human exposure through contaminated environments | Increased risk of exposure to antimicrobial resistant infections |

**Appendix 2:** Comparison of human and environmental surveillance approaches relevant to monitoring pathogens and antimicrobial resistance associated with plastic waste, including their scale of analysis, health outcomes monitored, and ability to measure exposure risk.

| **Surveillance method** | **Primary data source** | **Scale of analysis** | **Human health outcome monitored** | **Measures exposure risk?** | **Spatial information** | **Main strengths** | **Main limitations** |
| --- | --- | --- | --- | --- | --- | --- | --- |
| Passive clinical surveillance | Patients presenting to health facilities; laboratory-confirmed infections | Individual cases aggregated to health facility, district, or national level | Clinical disease and laboratory-confirmed infections | Indirectly, through diagnosed infections | Usually coarse; patient residence often unavailable or inconsistently recorded | Long-term routine surveillance; detects disease trends | Misses asymptomatic infections; dependent on healthcare seeking; limited fine-scale spatial resolution |
| Active community surveillance | Household surveys with collection of blood, stool or urine samples | Individual and household level | Clinical and subclinical infections | Yes | High, especially when GPS coordinates are collected | Captures infections missed by health facilities; enables spatial epidemiology | Expensive and labour intensive; usually limited in duration and coverage |
| Serological surveillance | Blood samples measuring pathogen-specific antibodies | Individual and community level | Previous and recent exposure, including asymptomatic infection | Yes | High when participant locations are recorded | Detects cumulative exposure; multiple pathogens can be measured simultaneously | Cannot always distinguish current from past infection; antibody persistence varies by pathogen |
| Environmental microbiological surveillance | Samples from waste piles, soil, rivers, wastewater, animals or surface water | Environmental sites | Environmental occurrence of pathogens rather than human disease | Indicates environmental exposure hazards | Sampling location level | Relatively inexpensive; identifies circulating pathogens and antimicrobial resistance; fewer ethical constraints | Does not directly measure human infection or identify transmission pathways |
| Wastewater surveillance | Wastewater, sewer networks, pit latrines or open drains | Community or catchment level | Community infection trends | Yes, at population level | Catchment or drainage network | Cost-effective early warning; useful for monitoring outbreaks | Requires wastewater infrastructure or adapted sampling; cannot identify infected individuals |
| Genomic surveillance | Whole genome sequencing of bacterial isolates from humans, animals and environment | Laboratory | Transmission relationships and antimicrobial resistance | Indirectly | Usually linked to sampling locations | High-resolution strain comparison; identifies shared populations | Direction of transmission often cannot be determined; resource intensive |
| Remote sensing supported environmental surveillance | Satellite or drone imagery combined with field sampling | Neighborhood to city scale | Does not measure disease directly | Identifies environmental exposure hazards | Very high spatial coverage | Repeated mapping of waste accumulation and dispersal; identifies potential environmental reservoirs | Detects waste rather than pathogens directly |
